# *Moringa* Genus: A Review of Phytochemistry and Pharmacology

**DOI:** 10.3389/fphar.2018.00108

**Published:** 2018-02-16

**Authors:** Nur Zahirah Abd Rani, Khairana Husain, Endang Kumolosasi

**Affiliations:** Drug and Herbal Research Centre, Faculty of Pharmacy, Universiti Kebangsaan Malaysia, Kuala Lumpur, Malaysia

**Keywords:** *Moringaceae*, *Moringa*, glucosinolates, pharmacological activity, phytochemistry

## Abstract

*Moringa* is a genus of medicinal plants that has been used traditionally to cure wounds and various diseases such as colds and diabetes. In addition, the genus is also consumed as a source of nutrients and widely used for purifying water. The genus consists of 13 species that have been widely cultivated throughout Asia and Africa for their multiple uses. The purpose of this review is to provide updated and categorized information on the traditional uses, phytochemistry, biological activities, and toxicological research of *Moringa* species in order to explore their therapeutic potential and evaluate future research opportunities. The literature reviewed for this paper was obtained from PubMed, ScienceDirect, and Google Scholar journal papers published from 1983 to March 2017. *Moringa* species are well-known for their antioxidant, anti-inflammatory, anticancer, and antihyperglycemic activities. Most of their biological activity is caused by their high content of flavonoids, glucosides, and glucosinolates. By documenting the traditional uses and biological activities of *Moringa* species, we hope to support new research on these plants, especially on those species whose biological properties have not been studied to date.

## Introduction

The genus *Moringa* is one of the genera found in the Moringaceae family along with *Anoma* and *Hyperanthera*. It is well-known as the “drumstick” or “horseradish” family. The *Moringa* genus comprises 13 species distributed through southwest Asia, southwest Africa, northeast Africa, and Madagascar. The species and their distributions are listed in Table 1.

**Table 1:** List of *Moringa* species throughout the world.

**Species**	**Country**	**Trivial name**
*M. arborea* Verdcourt	Kenya, Somalia	–
*M. borziana* Mattei	Kenya, Somalia	–
*M. concanensis* Nimmo	India	–
*M. drouhardii* Jumelle	Southern Madagascar	–
*M. hildebrandtii* Engler	Southwest Madagascar	Hildebrandt's Moringa
*M. longituba* Engler	Kenya, Southeast Ethiopia, Somalia	*Moringa tubiflora*
*M. oleifera* Lam.	India	Horseradish, Ben-oil Drumstick, Kelor
*M. ovalifolia* Dinter ex Berger	Namibia, Southwest Angola	Phantom Tree, Ghost Tree, African Moringo
*M. peregrina* Forssk. Ex Fiori	Red Sea, Arabia, Northeast Africa	Ben tree, wispy-needled Yasar tree, Wild drumstick tree, Yusor, Al Yassar, Al Ban
*M. pygmaea* Verdcourt	North Somalia	
*M. rivae* Chiovenda	Kenya, Ethiopia	Swanjehro
*M. ruspoliana* Engler	Kenya, Ethiopia, Somalia	
*M. stenopetala* (Baker f.) Cufodontis	Kenya, Southwest Ethiopia, Somalia	Cabbage tree, Haleko, Shelagda, Shiferaw

*All listed species were validated taxonomically from The Plant List (www.theplantlist.org, V1.1, 2013), Bihrmann's Caudiciforms (www.bihrmann.com)*.

Among the 13 species, current research is limited to *Moringa oleifera, Moringa stenopetala, Moringa concanensis*, and *Moringa peregrina*. As the other species are endemic to Madagascar and Northeast Africa, they are being evaluated less as there is less exploration for naturally occurring bioactive substances in these locations. In contrast, *M. oleifera*, which is native to India, is being studied widely. As a result, the species has been cultivated throughout the world, specifically in Asia, Latin America, Florida, the Caribbean, and the Pacific Islands (Fahey, 2005).

The species in this genus can be categorized into three groups depending on their type of trunk (Olson and Rosell, 2006). *M. stenopetala, Moringa drouhardii, Moringa ovalifolia*, and *Moringa hildebrandtii* have bloated water-storing trunks and are known as bottle trees. Meanwhile, *M. peregrina, M. concanensis*, and *M. oleifera* have slender trunks. The remaining species are tuberous shrubs endemic to Northeast Africa. *Moringa* species are also resistant to drought, and can grow fast without needing much care.

The *Moringa* genus has traditionally been widely used to improve health. Kings and queens used *Moringa* to improve their alertness and to maintain healthy skin. Indian warriors were fed *M. oleifera* leaves to enhance their energy and help to relieve their pain and stress during war (Mahmood et al., 2010). Other traditional uses of the genus are in healing skin infections, anxiety, asthma, wounds, fever, diarrhea, and sore throats.

The genus is well-known for its multiple uses. The seeds are used for purifying water, the leaves as nutrition supplements, the oil as a biofuel, the trunks as gum, the flowers as honey, and all of the plant parts can also be used for medicinal purposes (Fahey, 2005). *M. oleifera*, which is also known as the “Miracle Tree” and “Mother's Best Friend,” has been named the most nutrient-rich plant. Other than having a high concentration of vitamin A, vitamin C, potassium, and calcium, the plant contains all the essential amino acids (Mahmood et al., 2010).

Various research has been conducted on this genus to study its biological properties, especially on *M. oleifera* that has been under study since the 1970s (Mahmood et al., 2010). Currently, it is well-known that the plant has anti-inflammatory, antioxidant, anticancer, and antidiabetic activities. Recently, more research has been conducted on other species such as *M. concanensis, M. stenopetala*, and *M. peregrina*. However, no profound research on other species has been found. This review will primarily compile all the traditional uses, phytochemical contents, and biological activities of the *Moringa* genus, aiming to encourage new research on other species.

## Traditional uses

All the different parts of *Moringa* plants have been reported to have medicinal values. The traditional medicinal uses of *Moringa* species are tabulated in Table 2. Other than the species listed in Table 2, *Moringa arborea, Moringa longituba, Moringa borziana, Moringa pygmaea*, and *M. hildebrandtii* have also been reported to have medicinal value but have not been mentioned in connection with any specific activity. *Moringa* species are highly nutritious which benefits people in terms of providing daily nutritional supplements and boosting their immune systems (Popoola and Obembe, 2013). Mahmood et al. (2010) reported that *Moringa* leaves contain vitamin C, vitamin A, and high concentrations of essential amino acids. In addition, because the species are resistant to drought, *Moringa* species become valuable during the dry season when other vegetables are not available. In fact, *M. stenopetala* can be found in all households in the Gamo Gofa zone (Seifu, 2014). Mathur (2005) stated in his book that *Moringa* leaves are highly nutritious, with two times more protein than yogurt, four times more vitamin A than carrots, three times more potassium than bananas, seven times more vitamin C than oranges, and four times more calcium than milk. Zaghloul et al. (2012) reported that *M. peregrina* was also used as fodder to increase animal weight while *M. oleifera* contains high amount of zeatin that has been used as a natural plant growth enhancer and helps to increase crop yields (Leone et al., 2015b).

**Table 2:** Ethnomedicine of *Moringa* species.

**Species**	**Part**	**Traditional uses**	**References**
*M. concanensis*	Bark	Reduce pain, abortifacient	Patil and Patil, 2005
	Leaves	External tumors	Chitravadivu et al., 2009
	Resin	Fire burn wounds	
*M. drouhardii*	Bark	Colds and coughs	Olson, 1999
*M. peregrina*	Leaves	Skin rashes, paralysis	Odee et al., 2002
	Bark	Disinfectant to speed up wound healing	Marwah et al., 2007
	Pods	Infantile paralysis or convulsions	Miller et al., 1988
	Leaves, roots	Malaria, hypertension, stomach disorder, expel retained placenta, asthma, diabetes	Mekonnen et al., 1999
*M. rivae*	Leaves	Weakness of thigh and calf muscles	Forest Department, 2016
	Gum	Arthritis	
*M. ruspoliana*	–	Eye and throat infections, tsetse fly bites, livestock diseases, abdominal pains, sexually transmitted diseases	Odee et al., 2002
*M. stenopetala*	Leaves	Flu	Teklehaymanot and Giday, 2010
	Leaves	Diabetes and disorders associated	Habtemariam, 2016
	Root	Malaria, stomach pain, diabetes	Mekonnen, 2002
	Leaves	Malaria, hypertension, expel retained placenta, stomach pain, visceral leishmanial, diabetes, wound healing, common cold	
	Bark	Cough	Teklehaymanot and Giday, 2010
	Root	Epilepsy, help during labor	
*M. oleifera*	Leaf	Diarrheal, dysentery, colitis, sores, skin infection, anemia, cuts, scrapes, rashes, sign of aging	Silver, 2017
	Gum	Fevers, dysentery, asthma, dental decay	
	Seeds	Warts	
	Leaves	Antibacterial, antimalarial	Parrotta, 1993
	Oil	Gout, acute rheumatism	
	Flowers	Tumor, inflammation, hysteria, enlargement of spleen, muscle diseases, aphrodisiac substances	Anwar et al., 2007; Yabesh et al., 2014
	Roots	Toothache, anthelmintic, ant paralytic	Anwar et al., 2007; Popoola and Obembe, 2013; Sivasankari et al., 2014
	Barks	Aiding digestion, stomach pain, poor vision, ulcer, hypertension, joint pain, anemia, diabetes	Popoola and Obembe, 2013; Yabesh et al., 2014
	Leaves	Cardiac stimulants, malaria, arthritis, diseases of the skin, hypertension, typhoid fevers, swellings, parasitic diseases, diabetes, cuts, contraceptive remedy, genio-urinary ailments, boost immune system, elicit lactation	Anwar et al., 2007; Kasolo et al., 2010; Abe and Ohtani, 2013; Popoola and Obembe, 2013; Sivasankari et al., 2014; Yabesh et al., 2014

As well as their medicinal uses, *M. drouhardii, M. longituba, M. peregrina, M. stenopetala*, and *M. oleifera* have been used as coagulants to help clarifying water in addition to having antimicrobial activity (Bazrafshan et al., 2012; Dalvand et al., 2016). The high oleic acid content and high stability of *M. ovalifolia, M. stenopetala*, and *M. oleifera* seed oil makes it suitable to use as edible oil, cosmetic oil, biodiesel, and lubrication oil for machinery and watches (Rashid et al., 2008). Other than that, *M. stenopetala* is also used to expel snakes (Seifu, 2014), while the unique stature of *M. hildebrandtii* with its large trunk, and leaves and its scented flowers, is used to mark special occasions. It is also planted around the graves of the Mahabo tribe (Olson and Razafimmandimbison, 2000).

## Phytochemistry

**Figure 1:**
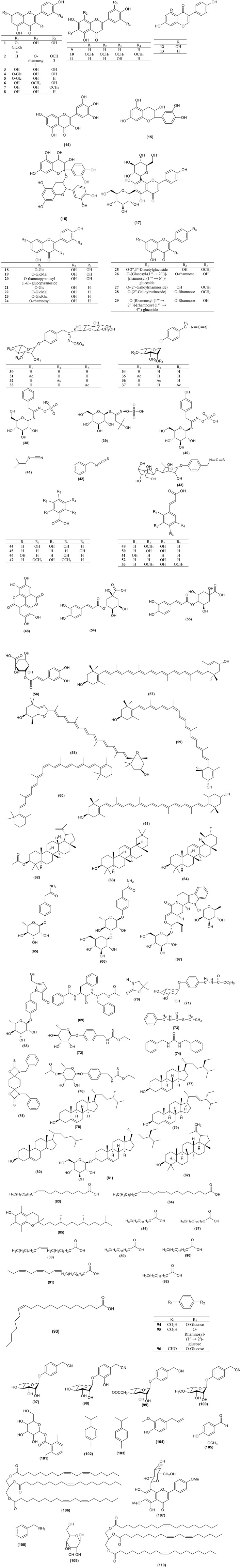
Structures of compounds from *Moringa* species.

*Moringa* species contain various phytoconstituents such as alkaloids, saponins, tannins, steroids, phenolic acids, glucosinolates, flavonoids, and terpenes. The diversity of these phytochemicals in the genus contributes to its numerous pharmacological uses. About 110 compounds were identified from the genus and are tabulated in Figure 1 and Table 3. Some of these compounds showed positive results when tested for various biological activities. In addition to these 110 compounds, the genus contains more compounds as detected by GC-MS. Regardless of the high phytochemical contents of the genus, the constituents of only specific species had been explored, namely *M. concanensis, M. peregrina, M. stenopetala*, and *M. oleifera*, and most of the studies focused on the leaves of the plants.

**Table 3:** Chemical constituents of *Moringa* species.

**No**.	**Compound name**	**Species**	**Part**	**References**
**FLAVANOIDS AND FLAVANOL GLYCOSIDES**				
**1**	Rutin	*M. stenopetala*, *M. peregrina*, *M. oleifera*	Leaves, aerial, leaves	Elbatran et al., 2005; Manguro and Lemmen, 2007; Devaraj et al., 2011; El-Alfy et al., 2011; Habtemariam and Varghese, 2015; Leone et al., 2015b
**2**	Chryseriol-7-*O*-rhamnoside	*M. peregrina*	Aerial	Elbatran et al., 2005; El-Alfy et al., 2011
**3**	Quercetin	*M. peregrina*, *M. oleifera*	Aerial, leaves	Elbatran et al., 2005; Devaraj et al., 2011; El-Alfy et al., 2011; Leone et al., 2015b
**4**	Isoquercetin	*M. oleifera*	Leaves	Vongsak et al., 2014
**5**	Astragalin	*M. oleifera*	Leaves	Vongsak et al., 2014
**6**	Rhamnetin	*M. peregrina*	Aerial	El-Alfy et al., 2011
**7**	Isorhamnetin	*M. oleifera*	Leaves	Leone et al., 2015b
**8**	Kaempferol	*M. oleifera*	Leaves	Manguro and Lemmen, 2007; Devaraj et al., 2011; Leone et al., 2015b
**9**	Apigenin	*M. oleifera*, *M. peregrina*	Leaves, aerial	Abdel-Rahman Tahany et al., 2010; El-Alfy et al., 2011; Leone et al., 2015b
**10**	6,8,3,5-Tetramethoxyapigenin	*M. peregrina*	Aerial	Elbatran et al., 2005
**11**	Luteolin	*M. oleifera*	Leaves	Leone et al., 2015a
**12**	Genistein	*M. oleifera*	Leaves	Leone et al., 2015a
**13**	Daidzein	*M. oleifera*	leaves	Leone et al., 2015a
**14**	Myricetin	*M. oleifera*	Leaves, seed	Lalas and Tsaknis, 2002; Leone et al., 2015a
**15**	Epicatechin	*M. oleifera*	Leaves	Leone et al., 2015a
**16**	Procyanidins	*M. oleifera*	Root, stem barks	Atawodi et al., 2010
**17**	Vicenin-2	*M. oleifera*	Leaves	Muhammad et al., 2016
**18**	Quercetin-3-*O*-glucoside	*M. oleifera*	Leaves, seeds	Manguro and Lemmen, 2007; Leone et al., 2015b; Maiyo et al., 2016
**19**	Quercetin-3-*O*-(6″-malonyl) glucoside	*M. oleifera*	Leaves	Leone et al., 2015a
**20**	Quercetin 3-*O*-α-*L*-rhamnopyranosyl(1-6)-β-*D*-glucopyranoside	*M. peregrina*		Dehshahri et al., 2012b
**21**	Kaempferol-3-*O*-glucoside	*M. oleifera*	Leaves	Leone et al., 2015a
**22**	Kaempferol-3-*O*-(6″-malonyl) glucoside	*M. oleifera*	Leaves	Leone et al., 2015a
**23**	Kaempferol-3-rutinoside	*M. oleifera*	Leaves	Leone et al., 2015a
**24**	Kaempherol-3-*O*-α-rhamnoside	*M. oleifera*	Leaves	Manguro and Lemmen, 2007
**25**	Kaempferide 3-*O*-(2″,3″-diacetylglucoside)	*M. oleifera*	Leaves	Manguro and Lemmen, 2007
**26**	Kaempferol-3-*O*-[β-glucosyl-(1 → 2)]-[α-rhamnosyl-(1 → 6)]-β-glucoside-7-*O*-α-rhamnoside	*M. oleifera*	Leaves	Manguro and Lemmen, 2007
**27**	Kaempferide-3-*O*-(2″-*O*-galloylrhamnoside)	*M. oleifera*	Leaves	Manguro and Lemmen, 2007
**28**	Kaempferide-3-*O*-(2″-*O*-galloylrutinoside)-7-*O*-α-rhamnoside	*M. oleifera*	Leaves	Manguro and Lemmen, 2007
**29**	Kaempferol-3-*O*-[α-rhamnosyl-(1 → 2)]-[α-rhamnosyl-(1 → 4)]β-glucoside-7- *O*-α-rhamnoside	*M. oleifera*	Leaves	Manguro and Lemmen, 2007
**GLUCOSINOLATE AND ISOTHIOCYANATE**				
**30**	4-(α-*L*-rhamnopyranosyloxy) benzyl glucosinolate (glucomoringin)	*M. oleifera*, *M. stenopetala*	Leaves, seed	Mekonnen and Drager, 2003; Leone et al., 2015b; Tumer et al., 2015
**31**	4-[(2′-*O*-acetyl-α-*L*-rhamnosyloxy) benzyl] Glucosinolate	*M. oleifera*	Leaves	Leone et al., 2015b; Tumer et al., 2015
**32**	4-[(3′-*O*-acetyl-α-*L*-rhamnosyloxy) benzyl] Glucosinolate	*M. oleifera*	Leaves	Leone et al., 2015b; Tumer et al., 2015
**33**	4-[(4′-*O*-Acetyl-α-*L*-rhamnosyloxy)benzyl] Glucosinolate	*M. oleifera*	Leaves	Leone et al., 2015b; Tumer et al., 2015
**34**	4-[(α-*L*-rhamnosyloxy) benzyl] Isothiocyanate	*M. oleifera*, *M. peregrina*	Leaves	Tumer et al., 2015
**35**	4-[(2′-*O*-acetyl-α-*L*-rhamnosyloxy) benzyl] Isothiocyanate	*M. oleifera*	Leaves	Tumer et al., 2015
**36**	4-[(3′-*O*-acetyl-α-*L*-rhamnosyloxy) benzyl] Isothiocyanate	*M. oleifera*	Leaves	Tumer et al., 2015
**37**	4-[(4′-*O*-acetyl-α-*L*-rhamnosyloxy) benzyl] Isothiocyanate	*M. oleifera*, *M. stenopetala*	Leaves	Mekonen and Gebreyesus, 2000; Tumer et al., 2015
**38**	Benzyl glucosinolate (glucotropaeolin)	*M. oleifera*	Seed	Saini et al., 2016
**39**	Glucoconringiin	*M. stenopetala*	Seed, leaves, roots	Mekonnen and Drager, 2003
**40**	Sinalbin	*M. oleifera*	Leaves	Leone et al., 2015b
**41**	Isobutylthiocyanate/Isothiocyanate	*M. stenopetala*, *M. peregrina*	Seed oil, stem, seed	Nibret and Wink, 2010; Dehshahri et al., 2012a
**42**	Benzyl isothiocyanate	*M. stenopetala*	Seed oil	Nibret and Wink, 2010
**43**	4-[(β-*D*-glucopyranosyl-1->4-α-*L*-rhamnopyranosyloxy) benzyl] Isothiocyanate	*M. oleifera*	Leaves, seeds	Maiyo et al., 2016
**PHENOLIC ACID**				
**44**	Gallic acid	*M. oleifera*	Leaves	Verma et al., 2009
**45**	Salicylic acid	*M. oleifera*	Leaves	Leone et al., 2015a
**46**	Gentisic acid	*M. oleifera*	Leaves	Leone et al., 2015a
**47**	Syringic acid	*M. oleifera*	Leaves	Leone et al., 2015a
**48**	Ellagic acid	*M. oleifera*	Leaves	Verma et al., 2009; Leone et al., 2015a
**49**	Ferulic acid	*M. oleifera*	Leaves	Verma et al., 2009; Leone et al., 2015a
**50**	Caffeic acid	*M. oleifera*	Leaves	Leone et al., 2015a
**51**	*o*-Coumaric acid	*M. oleifera*	Leaves	Leone et al., 2015a
**52**	*p*-Coumaric acid	*M. oleifera*	Leaves	Leone et al., 2015a
**53**	Sinapic acid	*M. oleifera*	Leaves	Leone et al., 2015a
**54**	Chlorogenic acid	*M. oleifera*,	Leaves	Verma et al., 2009; Leone et al., 2015b
**55**	Cryptochlorogenic acid	*M. oleifera*	Leaves	Vongsak et al., 2014
**56**	Neochlorogenic acid	*M. peregrina*	Aerial	El-Alfy et al., 2011
**Terpene**				
**57**	All-*E*-lutein	*M. oleifera*	Pods	Teixera et al., 2014; Saini et al., 2016
**58**	All-*E*-luteoxanthin	*M. oleifera*	Pods	Saini et al., 2016
**59**	13-*z*-Lutein	*M. oleifera*	Pods	Saini et al., 2016
**60**	15-*z-β*-Carotene	*M. oleifera*	Pods	Saini et al., 2016
**61**	All-*E*-Zeaxanthin	*M. oleifera*	Pods	Saini et al., 2016
**63**	β-Amyrin	*M. peregrina*	Aerial	El-Alfy et al., 2011
**64**	α-Amyrin	*M. peregrina*	Aerial	El-Alfy et al., 2011
**Alkaloid and Sterol**				
**65**	4′-hydroxyphenylethanamide-α-*L*-rhamnopyranoside (marumoside A)	*M. oleifera*	Leaves	Sahakitpichan et al., 2011
**66**	3″-*O*-β-*D*-glucopyranosyl derivatives (marumoside B)	*M. oleifera*	Leaves	Sahakitpichan et al., 2011
**67**	*N, α*-*L*-Rhamnopyranosyl vincosamide	*M. oleifera*	Leaves	Panda et al., 2013
**68**	Pyrrolemarumine-4″-*O*-α-*L*-rhamnopyranoside	*M. oleifera*	Leaves	Sahakitpichan et al., 2011
**69**	Aurantiamide acetate	*M. oleifera*	Roots	Sashidara et al., 2009
**70**	5,5-Dimethyloxazolidine-2-thione	*M. stenopetala*	Seeds	Mekonnen and Drager, 2003
**71**	*O*-Ethyl-4-[(α-*L*-rhamnosyloxy)-benzyl] carbamate	*M. oleifera*	Seeds	Guevara et al., 1999
**72**	Niazimicin	*M. oleifera*	Leaves, seeds	Guevara et al., 1999; Jung, 2014
**73**	*N*-benzyl, S-ethylthioformate	*M. oleifera*	Root bark	Nikkon et al., 2003
**74**	1, 3-Dibenzyl urea	*M. oleifera*	Roots	Sashidara et al., 2009
**75**	Pterygospermin	*M. oleifera*	Seeds	Das et al., 1957
**76**	Niaziminin	*M. oleifera*	Leaves	Murakami et al., 1998
**77**	β-sitosterol	*M. peregrina*, *M. oleifera*	Oil, aerial, leaves, seed	Abdel-Rahman Tahany et al., 2010; El-Alfy et al., 2011; Abd El Baky and El-Baroty, 2013; Maiyo et al., 2016
**78**	Campesterol	*M. peregrina*,	Oil	Abd El Baky and El-Baroty, 2013
**79**	Stigmasterol	*M. peregrina*,	Oil	Abd El Baky and El-Baroty, 2013
**80**	Cholest-5-en-3-ol	*M. stenopetala*	Root	Tesemma et al., 2013
**81**	β-Sitosterol-3-*O*-β-*D*-galactopyranoside	*M. oleifera*, *M. peregrina*	Stem bark	Abdel-Rahman Tahany et al., 2010; Bargah and Das, 2014
**82**	Lupeol	*M. peregrina*	Leaves	Safaeian et al., 2015
**OTHERS**				
**83**	Oleic acid	*M. oleifera*, *M. drouhardii*, *M. ovalifolia*, *M. peregrina*, *M. stenopetala*, *M. concanensis*, *M. hildebrandtii*	Oil, oil, oil	Kleiman et al., 2008; Nibret and Wink, 2010; Gaikwad et al., 2011; Abd El Baky and El-Baroty, 2013
**84**	Linoleic acid	*M. oleifera*, *M. drouhardii*, *M. ovalifolia*, *M. peregrina*, *M. stenopetala*, *M. concanensis*, *M. hildebrandtii*	Oil	Kleiman et al., 2008; Abd El Baky and El-Baroty, 2013
**85**	Tocopherols	*M. concanensis*	Leaves	Vijayakumar and Sumathi, 2016
**86**	Myristic acid	*M. oleifera*, *M. drouhardii*, *M. ovalifolia*, *M. peregrina*, *M. stenopetala*, *M. hildebrandtii*	Oil	Kleiman et al., 2008; Nibret and Wink, 2010; Abd El Baky and El-Baroty, 2013
**87**	Palmitic acid	*M. oleifera*, *M. drouhardii*, *M. ovalifolia*, *M. peregrina*, *M. stenopetala*, *M. concanensis*, *M. hildebrandtii*	Oil, roots	Kleiman et al., 2008; Nibret and Wink, 2010; Abd El Baky and El-Baroty, 2013; Faizi et al., 2014
**88**	Palmitoleic acid	*M. oleifera*, *M. drouhardii*, *M. ovalifolia*, *M. peregrina*, *M. stenopetala*, *M. concanensis*	Oil	Abd El Baky and El-Baroty, 2013
**89**	Stearic acid	*M. oleifera*, *M. drouhardii*, *M. ovalifolia*, *M. peregrina*, *M. stenopetala*, *M. concanensis*, *M. hildebrandtii*	Oil	Kleiman et al., 2008; Abd El Baky and El-Baroty, 2013
**90**	Arachidic acid	*M. oleifera*, *M. drouhardii*, *M. ovalifolia*, *M. peregrina*, *M. stenopetala*, *M. concanensis, M. hildebrandtii*	Roots, oil	Kleiman et al., 2008; Abd El Baky and El-Baroty, 2013; Faizi et al., 2014
**91**	Linolenic acid	*M. oleifera*, *M. drouhardii*, *M. ovalifolia*, *M. peregrina*, *M. stenopetala*	Oil	Abd El Baky and El-Baroty, 2013
**92**	Behenic acid	*M. oleifera*, *M. drouhardii*, *M. ovalifolia*, *M. peregrina*, *M. stenopetala*, *M. concanensis, M. hildebrandtii*	Oil	Kleiman et al., 2008; Abd El Baky and El-Baroty, 2013
**93**	Paullinic acid	*M. oleifera*, *M. drouhardii*, *M. ovalifolia*, *M. peregrina*, *M. stenopetala*, *M. concanensis*, *M. hildebrandtii*	Oil	Kleiman et al., 2008; Abd El Baky and El-Baroty, 2013
**94**	Benzoic acid 4-*O*-β-glucoside	M. oleifera	Leaves	Manguro and Lemmen, 2007
**95**	Benzoic acid 4-*O*-α-rhamnosyl-(1 → 2)-β-glucoside	*M. oleifera*	Leaves	Manguro and Lemmen, 2007
**96**	Benzaldehyde 4-*O*-β-glucoside	*M. oleifera*	Leaves	Manguro and Lemmen, 2007
**97**	Niazirin	*M. peregrina*, *M. oleifera*	Seeds, leaves	Faizi et al., 1994; Shanker et al., 2007; Sahakitpichan et al., 2011
**98**	Niaziridin	*M. oleifera*	Pods, leaves	Shanker et al., 2007
**99**	Niazirinin	*M. oleifera*	Leaves	Faizi et al., 1994
**100**	4-(4′-*O*-methyl-α-*L*-rhamnosyloxy) benzyl nitrile	*M. peregrina*	Seeds	El-Haddad et al., 2002
**101**	*M*oringyne	*M. oleifera*	Seeds	Memon et al., 1985
**102**	α-Phellandrene	*M. oleifera*	Oil	Ogunbino et al., 2009
**103**	*p*-Cymene	*M. peregrina*, *M. oleifera*	Seed, oil	Ogunbino et al., 2009; Dehshahri et al., 2012a
**104**	Eugenol	*M. oleifera*	Bark	Al-Asmari et al., 2015
**105**	Vanillin	*M. oleifera*	Leaves, fruits, seed	Singh et al., 2009
**106**	1,3-Dilinoleoyl-2-olein	*M. stenopetala*	Roots	Bekele et al., 2013
**107**	6-Methoxy-acacetin-8-C-β-glucoside	*M. peregrina*	Aerial	El-Alfy et al., 2011
**108**	Benzylamine	*M. oleifera*		Iffiu-Soltesz et al., 2010
**109**	*D*-allose	*M. oleifera*	Leaves	Al-Asmari et al., 2015
**110**	1,3-Dioleoyl-2-linolein	*M. stenopetala*	Roots	Bekele et al., 2013

### Flavonoids

The *Moringa* genus has high antioxidant activity mainly due to its high content of flavonoids. Most of the flavonoids present in the genus are in the flavanol and glycoside form. The most common flavonoids of the genus are rutin (**1**), quercetin (**3**), rhamnetin (**6**), kaempferol (**8**), apigenin (**9**), and myricetin (**14**). Optimization research has been conducted to discover the best way to extract flavonoids from *M. oleifera* with highest yield. As a result, subcritical ethanol extraction yielded 26.7% more flavonoid than a reflux method (Wang et al., 2017).

### Glucosinolate

*Moringa* species contain abundant glucosinolates. The most abundant glucosinolate present in the species is 4-*O*-(α-*L*-rhamnopyranosyloxy)-benzyl glucosinolate (**30**), also known as glucomoringin (GMG). Three isomers of 4-*O*-(α-*L*-acetylrhamnopyrosyloxy)-benzyl glucosinolate (**31–33**) were also detected in *M. oleifera* leaves, depending on the maturity and physiological properties of the leaves (Leone et al., 2015b).

Disruption of plant tissues usually from cutting or chewing caused the release of myrosinase which, when in contact with glucosinolates produces isothiocyanates. The most abundant isothiocyanate found in the genus, 4-[(α-*L*-rhamnosyloxy) benzyl] isothiocyanate (GMG-ITC) (**34**), is derived from 4-*O*-(α-*L*-rhamnopyranosyloxy)-benzyl glucosinolate (**30**). Recently, isothiocyanates have become a major research interest of *Moringa* for their various biological activities such as their anticancer, antidiabetic, antimicrobial, and anti-inflammatory effect (Park et al., 2011; Padla et al., 2012; Waterman et al., 2014, 2015). The alkylation of isothiocyanates with proteins and DNA contribute to their biological activity (Nibret and Wink, 2010). Dehshahri et al. (2012a) initiated an *in vitro* callus culture study of *M. peregrina* to induce the production of isothiocyanates but the study did not manage to produce any. Isothiocyanate is commonly present as a volatile oil and is not stable at room temperature. In contrast, *M. oleifera's* isothiocyanate is very stable and present as a solid at room temperature because of the additional sugar moiety on its structure (Tumer et al., 2015).

### Phenolic acid

*M. oleifera* leaves contain gallic acid (**44**) as their major phenolic acid. Ellagic acid (**48**), ferulic acid (**49**), caffeic acid (**50**), *o*-coumaric acid (**51**), and chlorogenic acid (**54**), are also detected in the leaves and gentisic acid (**46**), syringic acid (**47**), ρ-coumaric acid (**52**), and sinapic acid (**53**) were detected in trace amounts (Leone et al., 2015a,b). Other phenolic acid constituents in the species are tabulated in Figure 1 and Table 3.

### Terpenes

Teixera et al. (2014) and Saini et al. (2014) reported that the major carotenoid detected in *M. oleifera* leaves is lutein (**57**). Saini et al. (2014) reported that *M. oleifera* did not contain α-carotene which can usually be found in green leafy plants. The author assumed that all of the α-carotene had been fully converted into lutein. Other carotenoids that can be found in the plant are all-*E*-luteoxanthin (**58**), 13-*Z*-lutein (**59**), 15-*Z*-β-carotene (**60**), and all-*E*-zeaxanthin (**61**) (Saini et al., 2014). Lupeol acetate (**62**), β-amyrin (**63**), and α-amyrin (**64**) were isolated from an *n*-hexane fraction of an ethanol extract of the aerial part of *M. peregrina* (El-Alfy et al., 2011).

### Alkaloids

Two new pyrrole alkaloid glycosides were isolated from *M. oleifera* leaves, marumoside A (**65**) and marumoside B (**66**) together with pyrrolemarumine-4″-*O*-α-*L*-rhamnopyranoside (**68**) (Sahakitpichan et al., 2011).

### Sterols

A sterol glycoside, namely β-sitosterol-3-*O*-β-*D*-galactopyranoside (**81**), was isolated from a chloroform extract of *M. oleifera* stem bark (Bargah and Das, 2014). The main steroidal components in *M. peregrina* oil were β-sitosterol (**77**) (56.76%), campesterol (**78**) (23.24%), and stigmasterol (**79**) (8.11%) (Abd El Baky and El-Baroty, 2013). β-sitosterol (**77**) was isolated from the leaves and seeds of *M. oleifera* (Maiyo et al., 2016) and an acetone extract of *M. stenopetala* root wood has been reported to contain cholest-5-en-3-ol (**80**) (Tesemma et al., 2013).

### Others

Shanker et al. (2007) reported that two nitrile glycosides, niazirin (**97**) and niaziridin (**98**), were observed by reverse phase HPLC. From the three parts of *M. oleifera* tested, only the leaves and pods showed peaks for these nitrile glycosides while no corresponding peaks were detected from the bark. The content of niazirin (**97**) was higher in the leaves while the pods contained a higher concentration of niaziridin (**98**). 6-methoxy-acacetin-8-C-β-glucoside (**107**) was first isolated from an ethanolic extract of the aerial part of *M. peregrina* (El-Alfy et al., 2011).

The major fatty acids of *M. peregrina* are oleic acid (18:1) (**83**) 65.36% and linoleic acid (18:3) (**84**) 15.32%. The oil also contains a high content of tocopherols (**85**) and phenols (Abd El Baky and El-Baroty, 2013). Oleic acid (**83**) (18:1), linoleic acid (**84**) (18:2), myristic acid (**86**) (14:0), palmitic acid (**87**) (16:0), palmitoleic acid (**88**) (16:1), stearic acid (**89**) (18:0), arachidic acid (**90**) (20:0), linolenic acid (**91**) (18:3), behenic acid (**92**) (22:0), and paullinic acid (**93**) (20:1) are present in *M. oleifera, M. drouhardii, M. ovalifolia, M. peregrina*, and *M. stenopetala. M. concanensis* contains all the fatty acids listed above except myristic acid (**86**) and linolenic acid (**91**). *M. hildebrandtii* also contains all the fatty acids listed except for palmitoleic acid (**88**) and linolenic acid (**91**) (Kleiman et al., 2008). The species contain high oleic acid (**83**) levels from 68 to 79%. However, the fatty acid content is dependent on the location where the oil is obtained. *M. oleifera* seed oil has a nutty flavor and has light yellow color (Nadeem and Imran, 2016). The oil contains a high concentration of oleic acid (**83**) which constitutes 75–77% of the fatty acid composition of the seeds.

## Biological activities

### Antioxidant

The high phenolic content of *Moringa* species contributes to their high antioxidant activity. Phenolic compounds stabilize radicals produced in cells by donating or accepting electrons, hence acting as antioxidants. A water extract of *M. stenopetala* leaves had higher DPPH (1,1-di-phenyl-2-picrylhydrazyl) inhibition (IC_50_: 40 μg/mL) than a similar extract of *M. oleifera* leaves (IC_50_: 215 μg/mL). Rutin (**1**) also possessed high antioxidant activity (IC_50_: 5 μg/mL) in a DPPH assay. An HPLC analysis showed a higher content of rutin (**1**) in *M. stenopetala*, making it a stronger antioxidant than *M. oleifera* (Habtemariam and Varghese, 2015). A methanol fraction of *M. peregrina* leaves showed DPPH inhibition (IC_50_: 17.07 μg/mL) which was comparable to ascorbic acid's DPPH inhibition (IC_50_: 13.68 μg/mL) (Al-Owaisi et al., 2014). Based on HPLC results, *M. peregrina* leaves' hexane fraction did not contain phenolic compounds, but did possess radical scavenging activity. Abd El Baky and El-Baroty (2013) reported that *M. peregrina* seed oil also had significant antioxidant activity compared to the common antioxidants BHA, α-tocopherol, and BHT. *M. ovalifolia*, specifically its bark, contained quercetin (**3)**, kaempferol (**8**), and myricetin (**14**) that showed antioxidant activity by increasing ferric reducing activity and inhibiting DPPH activity (Ananias, 2015). A study reported that pre-treatment of *M. peregrina* leaves could prevent the plasma hydrogen peroxide concentration from rising at doses of 200 and 400 mg/kg (Safaeian et al., 2015). It also reduced the elevated hydrogen peroxide concentration in plasma and increased the ferric reducing antioxidant at doses of 400 mg/kg. Santhi and Sengottuvel (2016) reported that a methanol extract of *M. concanensis* leaves inhibited DPPH activity, hydroxyl radicals, reducing power, and superoxide anion radicals. The hydroxyl radical inhibition of the extract (IC_50_: 45.3 μg/mL) was stronger than that of ascorbic acid (IC_50_: 58.2 μg/mL). Ndhlala et al. (2014) studied the phytochemical properties, and the antioxidant and antimicrobial activities of *M. oleifera* from 13 different cultivars around the world. It was found that *M. oleifera* from different cultivars had different antioxidant, phytochemical, and antimicrobial profiles. *M. oleifera* from one of Thailand's cultivars was five times stronger in inhibiting DPPH radical scavenging activity than ascorbic acid was.

Verma et al. (2009) reported that the most active fraction of a *M. oleifera* leaf hydromethanolic extract was the ethyl acetate fraction. The fraction inhibited DPPH by IC_50_ at 0.04 mg/mL, which was comparable with quercetin (**3**) activity that had inhibited DPPH with an IC_50_-value of 0.02 mg/mL. As well as *in-vitro* tests, the ethyl acetate fraction of *M. oleifera* leaves has been tested on CCl_4_ intoxicated rats (Verma et al., 2009). The extract increased superoxide dismutase (SOD), catalase (CAT), and reduced glutathione (GSH) levels. Pre-treatment of the *M. oleifera* leaf hydroethanolic extract also counteracted hepatoxicity induced by paracetamol in Sprague-Dawley rats by reducing lipid peroxidation levels and normalizing antioxidant enzyme levels (Uma et al., 2010). A study observed that *M. oleifera* leaf extract reduced DNA breakage in KB cells in addition to increasing their antioxidant enzymes and inhibiting lipid peroxidation (Sreelatha and Padma, 2011). The antioxidant activity of leaf, oil, and seed ethanolic extracts of *M. oleifera* have been reported to display renal protective and hepatoprotective activity against gamma radiation, HgCl_2_, acetaminophen, and arsenic (Gupta et al., 2007; Fakurazi et al., 2008; Sinha et al., 2011; Abarikwu et al., 2017). Agrawal et al. (2015) observed that *M. oleifera* hydro alcoholic root extract acted synergistically with curcumin and with piperine in inhibiting oxidative stress induced by beryllium in rats. By controlling GSH level, *M. oleifera* ethanol extract reduced glucose-induced cataractogenesis of isolated goat eye lenses (Kurmi et al., 2014). This activity was also observed in the flavonoid fraction of the *M. oleifera* leaves that reduced selenite-induced cataractogenesis in rat pups (Sasikala et al., 2010).

A study stated that myricetin (**14**) from *M. oleifera* seeds had stronger antioxidant activity than α*-*tocopherol and BHT (Lalas and Tsaknis, 2002). A leaf extract of *M. oleifera* contained isoquercetin (**4**), astragalin (**5)**, and crypto-chlorogenic acid (**54**). The leaf extract of the plant, together with the compounds, reduced reactive oxygen species in HEK-293 cells that were induced by H_2_O_2_ (Vongsak et al., 2015). The compound that had the highest antioxidant activity was determined to be isoquercetin (**4**) as it increased the mRNA expression levels of CAT, heme oxygenase 1, and SOD. Maiyo et al. (2016) isolated two compounds from *M. oleifera* seeds and leaves that showed antioxidant activity: quercetin-3-*O*-glucoside (**18**) displayed significant antioxidant activity while 4-(β-*D*-glucopyranosyl-1->4-α-*L*-rhamnopyranosyloxy) benzylisothiocyanate (**43**)'s activity was moderate.

Ngamukote et al. (2016) conducted a study on the effects of *M. oleifera* extract on the fasting plasma glucose (FPG) concentrations of healthy volunteers, in addition to its antioxidant activity. The extract reduced plasma malondialdehyde (MDA) levels, increased Trolox equivalent antioxidant activity, increased the ferric reducing ability of the plasma, and did not change the FPG concentration compared with that of healthy volunteers that were fed only warm water.

### Anti-convulsant

Experiments to discern the effects of *M. concanensis* leaf ethanol extract on the maximal electroshock seizure test and the pentylene tetrazole-induced convulsion test were conducted on Swiss albino mice (Joy et al., 2013). For both of the tests, *M. concanensis* inhibited mortality compared to control group in which deaths resulted. The study reported that the extract might block either calcium channels, sodium channels, or NMDA receptors, or has GABA agonist activity.

### Anticancer

The main pathway for the anticancer activity of *Moringa* species is by inhibiting proliferation through apoptosis. Table 4 summarizes the cancerous cell lines that have been inhibited by *Moringa* species. Methanol crude extracts of *M. concanensis* root bark inhibited the proliferation of hepatocellular carcinoma (Hep-G2) cells through intrinsic pathways by regulating caspase 9 and caspase 3 while reducing the mitochondrial membrane potential of the cells (Vijayarajan and Pandian, 2016). (4′-*O*-acetyl-α-*L*-rhamnopyranosyloxy)benzyl isothiocyanate (**37**) and niazimicin (**70**) were responsible for the regulation of caspase 9 activity (Tiloke et al., 2013). *M. oleifera* leaf extract decreased the proliferation of B16F10 melanoma cells in addition to causing roughly 22% cancerous cell death (Gismondi et al., 2013). It caused apoptosis at the sub G1-area and induced cell arrest at the G2/M phase. The extract increased the p27^Kip1^, p^53^, and p21^WAF1/Cip1^ levels of the cells. Moringin (**34**) inhibited malignant astrocytoma cells by oxidative stress-mediated apoptosis through Bax and p53 activation pathways (Rajan et al., 2016). Nibret and Wink (2010) reported that seed oil from *M. stenopetala* inhibited the proliferation of HL-60 cells with IC_50_: 11.63 μg/mL. The bioactive compound reported was benzyl isothiocyanate (**42**), that had high cytotoxic activity against HL-60 cell with IC_50_: 4.62 μg/mL.

**Table 4:** Cell line studied for anticancer activity of *Moringa* species.

**Species**	**Cancerous cell line inhibited**	**References**
*M. concanensis*	Hep G2	Vijayarajan and Pandian, 2016
*M. oleifera*	A549, Hep-G2, Panc-1, p34, COLO 357, MDA-MB-231, HCT-8, MCF-7, HeLa, CACO-2, L929, HCT-16, PC3, K562, THP-1, T47D, HL-60, Colo-205	Monera et al., 2008; Waiyaput et al., 2012; Berkovich et al., 2013; Tiloke et al., 2013; Al-Asmari et al., 2015; Diab et al., 2015; Elsayed et al., 2015; Jung et al., 2015; Madi et al., 2016
*M. peregrina*	MCF-7, Hep G2, HCT 116	El-Alfy et al., 2011; Abd El Baky and El-Baroty, 2013
*M. stenopetala*	HL-60, Hep-G2	Mekonnen et al., 2005; Nibret and Wink, 2010

An ethanol extract of *M. stenopetala* leaves and seeds reduced Hep-G2 activity and increased LDH leakage in a dose and time-dependent manner (Mekonnen et al., 2005). 4-(β-*D*-glucopyranosyl-1->4-α-rhamnopyranosyloxy)-benzylisothiocyanate (**43**) had stronger anticancer activity than quercetin-3-*O*-glucoside (**18**) against Caco-2 and Hep-G2 cells (Maiyo et al., 2016). The presence of eugenol (**101**) in *M. oleifera* bark inhibited the activity of E2F1/survivin and the presence of *D*-allose (**109**) in *M. oleifera* leaves inhibited cancer cells in the G1 phase of MDA-MB-231 and HCT-8 cells (Al-Asmari et al., 2015).

A water extract of *M. oleifera* pods exhibited suppressive effects on dextran sodium sulfate- and azoxymethane-induced mouse colon carcinogenesis (Budda et al., 2011). The extract reduced COX-2 proteins and iNOS expression in addition to reducing the PCNA index of the mice. The extract also reduced the multiplicity and incidence of the tumors. The study reported that the high content of omega-9 oleic fatty acid (**83**) in the extract, that possesses anti-inflammatory activity, could modulate this cell proliferation. Alternately, glucomoringin (**30**) might also be responsible for this antitumor activity.

A hydro alcoholic extract of *M. oleifera* also exhibited antitumorigenic activity by balancing xenobiotic metabolism between Phase I and Phase II (Bharali et al., 2003). The extract increased Cyt P450 and Cyt b5 activity in Phase I while increasing glutathione S-transferase, glutathione reductase, and glutathione peroxidase, and reducing the GSH levels that are responsible for Phase II. The study also reported that the extract might act as a “blocking agent” in reducing xenobiotic substrates for Phase II. In addition, the extract increased CAT concentration while reducing mouse skin papilloma genesis and lipid peroxidation.

### Antimicrobial

Various research has been conducted on *Moringa* species for their antimicrobial activity. Table 5 summarizes the antimicrobial activity of each species. *Moringa* species have been widely used as water purifiers and antiseptics for water treatment because of their high antimicrobial activity. Hexane and methanol seed extracts of both *M. oleifera* and *M. stenopetala* showed inhibition against waterborne pathogens, particularly against *Salmonella typhii, Vibrio cholera*, and *Escherichia coli* (Walter et al., 2011). Most of the extracts showed better inhibition in lower concentrations.

**Table 5:** Microorganisms studied for antimicrobial activity of *Moringa* species.

**Species**	**Microorganism inhibited**	**References**
*M. concanensis*	*Proteus vulgaris, Lactobacillus brevis, Pseudomonas* sp. *Staphylococcus* sp.*, Micrococcus luteus, Bacillus* sp.*, Lactobacillus bulgaricus, Aspergillus sojae, Aspergillus niger, Aspergillus oryzae, Aspergillus flavus, Candida albicans, Vibrio cholera, Escherichia coli, Klebsiella pneumoniae, Pseudomonas aeruginosa*	Balamurugan and Balakrishnan, 2013; Karmegam and Nagaraj, 2017
*M. oleifera*	*Aspergillus flavin, Trichoderma* sp.*, Staphylococcus aureus, Shigella dysenteriae, Shigella boydii, Bacillus megaterium, Escherichia coli, Enterobacter aerogenes, Providencia stuartii, Klebsiella pneumoniae, Pseudomonas aeruginosa, Bacillus subtilis, Mycobacterium phlei, Bacillus cereus, Sarcina lutea, Basidiobolus ranarum, Basidiobolus haptosporus, Propionibacterium acnes, Staph epidermis, Strep pyogenes, Trichophyton mentagrophytes, Microsporum canis, Trichophyton rubrum, Epidermophyton floccosum, Pseudomonas fluorescens, Citrobacter freundii, Serratia marcescens, Enterobacter* sp.*, Salmonella* sp.*, Proteus vulgaris, Streptococcus mutans, Vibrio parahaemolyticus, Enterococcus faecalis, Aeromonas cavieae, Streptococcus aureus, Streptococcus mutans, Penicillium expansum, Penicillium digitatum, Penicillium aurantiogriseum, Penicillium citrinum, Aspergillus niger spp., Microcytis aeruginosa, Salmonella typhii, Vibrio cholera, Candida albicans*	Nwosu and Okafor, 1995; Chuang et al., 2007; Lurling and Beekman, 2010; Rahman et al., 2010; Peixoto et al., 2011; Saadabi and Abu Zaid, 2011; Walter et al., 2011; Padla et al., 2012; Rattanasena, 2012; Galuppo et al., 2013; Marrufo et al., 2013; Patel et al., 2014; Zaffer et al., 2014; Eyarefe et al., 2015; Dzotam et al., 2016; Elgamily et al., 2016
*M. ovalifolia*	*Escherichia coli, Bacillus cereus, Enterococcus faecalis*	Shailemo et al., 2016
*M. peregrina*	*Escherichia coli, Staphylococcus aureus, Candida albicans, Pseudomonas aeruginosa, Staphylococcus epidermis, Candida glabrata, Klebsiella pneumoniae, Enterobacter cloacae, Candida tropicalis, Enterococcus* sp.*, Aeromonas hydrophila*	Abdel-Rahman Tahany et al., 2010; Lalas et al., 2012; El-Awady et al., 2015
*M. stenopetala*	*Salmonella typhii, Vibrio cholera, Escherichia coli, Shigella spp., Staphylococcus aureus, Candida albicans, Pseudomonad aeruginosa*	Walter et al., 2011; Tesemma et al., 2013; Seifu, 2014

Ethyl acetate, acetone, and ethanol extracts of *M. oleifera* seeds, roots, leaves, and a mixture, were assessed for their dental antibacterial and antifungal activity (Elgamily et al., 2016). All of the extracts showed inhibition of *Streptococcus aureus* and *Streptococcus mutans* with the ethanol extract and leaf extract showing the highest inhibition. In contrast, none of the extracts showed inhibition against *Candida albicans*. Another study reported that higher concentrations of *M. oleifera* seeds were needed to inhibit the growth of *C. albicans* (Saadabi and Abu Zaid, 2011). The ethanolic leaf extract of *M. oleifera* was formulated into mouthwash and toothpaste (Elgamily et al., 2016). As a result, the toothpaste showed inhibition of *Streptococcus aureus, S. mutans*, and *C. albicans*, but the mouthwash only showed antimicrobial activity. The ethanol extracts of the seeds and leaves of *M. oleifera* also exhibited inhibition against the dermatophytes *Trichophyton mentagrophytes, Microsporum canis, Trichophyton rubrum*, and *Epidermophyton floccosum* (Chuang et al., 2007).

Hexane, ethyl acetate, methanol, and chloroform extracts of *M. oleifera* leaves were also tested on different diarrhea associated bacteria: *Serratia marcescens, Shigella dysenteriae, Enterobacter* sp.*, E. coli, Klebsiella pneumoniae*, and *Salmonella* sp. (Rahman et al., 2010). All of the extracts exhibited antibacterial activity against the bacteria with minimum inhibitory concentrations ranging from 62.5 to 1,000 μg/mL and zones of inhibition of 8–23.2 mm. Peixoto et al. (2011) reported that aqueous and ethanolic extracts of *M. oleifera* leaves showed inhibition against *Staphylococcus aureus, Vibrio parahaemolyticus, Enterococcus faecalis*, and *Aeromonas caviae*. In contrast, the extracts showed negative result on *E. coli, Salmonella enteritidis*, and *Pseudomonas aeruginosa*. Throughout the study it was observed that the extract showed stronger inhibition against gram-positive species than gram-negative species.

The bioactive compounds isolated from *M. stenopetala* were cholest-5-en-3-ol (**80**), oleic acid (**83**), and palmitic acid (**87**) (Tesemma et al., 2013). From the compounds listed, cholest-5-en-3-ol (**80**) possessed the strongest antibacterial activity against *E. coli*. Abdel-Rahman Tahany et al. (2010) isolated lupeol acetate (**62**), β-amyrin (**63**), α-amyrin (**64**), β-sitosterol (**77**), and β-sitosterol-3-*O*-β-*D*-glucoside (**81**) from *M. peregrina* and these compounds showed higher inhibition toward bacteria than toward fungi. β-Sitosterol (**77**) and β-sitosterol-3-*O*-β-*D*-glucoside (**81**), particularly, only possessed antibacterial activity. It was also reported that *M. concanensis* showed a synergistic effect with *Hugonia mystax* and *Curcuma neilgherrensis* with better activity against *E. coli, P. aeruginosa*, and *K. pneumoniae* than when tested alone against the bacteria (Karmegam and Nagaraj, 2017).

Nikkon et al. (2003) isolated aglycon of deoxy-niazimicine (*N*-benzyl, *S*-ethyl thioformate) (**73**) from a chloroform extract of *M. oleifera* root bark and this compound showed stronger inhibition toward *Staphylococcus aureus, S. dysenteriae, Shigella boydii, S. typhii, P. aeruginosa, C. albicans*, and *Aspergillus flavus* growth than a crude extract. 4-[(α-*L*-rhamnosyloxy)-benzyl] isothiocyanate (**34**) from *M. oleifera* and *M. stenopetala* seeds had minimal bactericidal concentrations of 56 μmol/l for *Bacillus subtilis* and 40 μmol/l for *Mycobacterium phlei* (Eilert et al., 2007). This isothiocyanate, together with 4-[(4′-*O*-acetyl-α-*L*-rhamnosyloxy)-benzyl] isothiocyanate (**37**) from *M. oleifera* seeds, exhibited antimicrobial activity against *Staphylococcus aureus, Staphylococcus epidermis, B. subtilis, E. floccosum*, and *T. rubrum* (Padla et al., 2012). Zaffer et al. (2014) reported that an ethyl acetate extract of *M. oleifera* bark showed higher inhibition toward *Pseudomonas fluorescens, Staphylococcus aureus, Bacillus megaterium*, and *Citrobacter freundii* than methanol, chloroform, and aqueous extracts of the same part of the plant.

Torondel et al. (2014) reported the effectiveness of dried and wet *M. oleifera* leaf powder as a hand-washing product in healthy volunteers. The results showed that, only the highest dose of *M. oleifera*, 4 g, showed levels of inhibition of *E. coli* comparable to a non-medicated liquid soap. The study reported that this activity was not related to the mechanical friction produced while washing hands. As aqueous preparations of *M. oleifera* leaf powder exhibited stronger microbial inhibition than dried preparations, the study reported that this activity might be due to saponin presence in the extract that had surfactant properties.

### Antitrypanosomal

In the *Moringa* genus, *M. stenopetala* showed significant antitrypanosomal activity. *M. stenopetala* leaf acetone extract and root ethanol extract inhibited the infective stages of *Trypanosoma brucei* (Mekonnen, 2002). A methanol extract of *M. stenopetala* maintained 100% survival against *Trypanosoma congolense* (Kifleyohannes et al., 2014). It also minimized PCV which is a method of controlling anemia. Dichloromethane and methane extracts of the seeds had low trypanocidal activity while the essential oils that contain mainly benzyl isothiocyanate (**42**) and isobutyl isothiocyanate (**41**) showed potent trypanocidal activity (Nibret and Wink, 2010). Petroleum ether, chloroform, methanol, and aqueous fractions of *M. oleifera* leaves, stem bark, and roots inhibited *Trypanosoma brucei brucei* (Ibrahim et al., 2014). Ayyari et al. (2013) reported that 4-[(α-*L*-rhamnosyloxy) benzyl] isothiocyanate (**34**) from *M. peregrina* aerial parts inhibited *Trypanosoma brucei rhodesiense* with an IC_50_ of 0.1 μM. With a dose 10 mg/kg for four consecutive days, the compound reduced parasitemia by 95% but the parasitemia increased again after 10 days of infection. The compound was reported to have the potential to become a high quality antitrypanosomal drug as it inhibited the parasite at specific enzymes and irreversibly inhibited trypanothione reductase. An acetone extract of *M. stenopetala* leaves and an ethanol extract of *M. stenopetala* root wood inhibited *Trypanosoma brucei* trypomastigotes with ED_50_-values of 10 and 9.2 μg/mL, respectively (Mekonnen et al., 1999).

### Antileishmanial

Mekonnen and Gessesse (1998) reported that high concentrations of *M. stenopetala* extract, 600 to 1,500 μg/mL, changed *Leishmania donovani* promastigotes' shape and resulted in the loss to their flagella in 40–95% of them. A root extract of the plant inhibited *Leishmanial aethiopica* in mice (Bekele et al., 2013). 1,3-Dilinoleoyl-2-olein (**106**) and 1,3-dioleoyl-2-linolein (**110**) from the roots inhibited the promastigotes and amastigotes stages of the parasite. The activity of 1,3-Dilinoleoyl-2-olein (**106**) in inhibiting the promastigotes stage was comparable to that of the positive control, miltefosine.

Kaur et al. (2014) reported that a 70% ethanolic extract of *M. oleifera* roots and a methanolic extract of *M. oleifera* leaves displayed antileishmanial activity against *L. donovani* promastigotes. The ethyl acetate fraction of a methanolic extract inhibited leishmaniasis with an IC_50_ of 27.5 μg/mL. Niazinin, isolated from the ethyl acetate fraction, showed the most antileishmanial activity with an IC_50_ of 27.5 μg/mL. Singh et al. (2015) observed antileishmanial activity from different parts of *M. oleifera*, the bark, leaf, stem, flower, and root. The flower, especially from the ethyl acetate fraction, showed the most potent activity against *L. donovani* promastigotes in infected macrophages by inhibiting parasite viability in a dose- and time-dependent manner. The extract also reduced parasite activity in both the spleen and the liver of Balb/c mice.

### Antiviral

*M. oleifera* extract possessed antiviral activity against the herpes simplex virus type 1 (HSV-1) by inhibiting more than 50% of plaque formation at a 100 μg/mL dose (Lipipun et al., 2003). The extract inhibited phosphonoacetate-resistant HSV-1 and kinase-deficient HSV-1 strains in mice. At a dose of 750 mg/kg, the extract reduced the mortality of the infected mice by prolonging their mean survival time and delaying the development of skin lesions. An aqueous extract of *M. oleifera* leaves activated cellular immunity in mice that were infected with HSV-1 by reducing the virus concentration and limiting herpetic skin lesion development (Kurokawa et al., 2016). *M. peregrina* seed oil was also reported to have antiviral effects against HSV (Soltan and Zaki, 2009). Murakami et al. (1998) reported that 4-[(4′-*O*-acetyl-alpha-*L*-rhamnosyloxy) benzyl] isothiocyanate (**37**) and niaziminin (**76**) inhibited Epstein-Barr virus activation. *M. oleifera* also showed inhibition against the foot and mouth disease virus at concentrations of 1–50 μg/mL (Younus et al., 2016).

A buffer extract of *M. oleifera* fruits exhibited anti-HBV activity and a hydroalcoholic extract of the plant's leaves reduced the ccDNA level of HBV in HepG2 cells (Waiyaput et al., 2012). According to a survey, *M. oleifera* was being used as a supplement toward antiretroviral therapy for HIV infection, but no further research has been conducted on the efficiency of the plant as an antiviral substance (Monera and Maponga, 2010).

### Antihyperglycemic, antihyperlipidemic, and hypocholesterolemic

Ethanol and aqueous extracts of the aerial parts of *M. peregrina* exhibited antihyperglycemic activity against streptozotocin diabetic rats by lowering their blood glucose levels (El-Alfy et al., 2011). The hexane fraction from an ethanol extract of the plant reduced blood glucose levels by 64–77.44% for 3 h after 30 min of administration. The study suggested that this activity might be because the fraction contained the antihyperglycemic compounds lupeol acetate (**62**) and β-sitosterol (**77**). *M. stenopetala* leaf hydroalcoholic extract was reported to inhibit certain enzymes that related to hyperglycemia and hyperlipidia, such as maltase, sucrase, pancreatic cholesterol esterase, pancreatic lipase, and pancreatic α-amylase (Toma et al., 2014). The leaf extract also reduced cholesterol, triglycerides, and the glucose lipid profile in fructose-induced rats (Geleta et al., 2016b).

Sangkitikomol et al. (2014) evaluated the effects of *M. oleifera* leaf hydroethanolic extract on advanced glycation end products. Throughout the study, the extract reduced the mRNA expression of PPARα1, PPAR-γ, and HMG-CoAR that are responsible for maintaining lipid homeostasis. The extract inhibited the formation of AGE in HepG2 cells at doses of 2.5–10 mg/mL. *M. oleifera* aqueous leaf extract also inhibited formation of both non-fluorescent and fluorescent advanced glycation end products by reducing monosaccharides, in addition to reducing the oxidation of thiols and protein carbonyl content (Nunthanawanich et al., 2016).

A study reported that an aqueous extract of *M. oleifera* leaves increased insulin levels and decreased insulin resistance, helping to combat hyperglycemia in diabetic rats (Tuorkey, 2016). The extract reduced creatinine and urea levels from damaged kidneys and increased the immune tolerance of the diabetic rats by increasing the activity of CD69, INF-γ, and CD44. Insulin-like protein was observed in the seed coat of *M. oleifera* (Paula et al., 2016). This protein had antigenic epitopes similar to insulin and displayed hypoglycemic activity on oral administration. The same activity was observed by a protein from *M. oleifera* leaves (Paula et al., 2017). The protein reduced blood glucose levels after single and repeated doses of the extract. It displayed antioxidant activity by increasing CAT levels and reducing MDA levels. It also cross-reacted with anti-insulin antibodies which proved that it might have antigenic epitopes similar to insulin. *M. oleifera* leaf extract reduced FPG level, post-prandial levels, blood glycated hemoglobin, total cholesterol, non-HDL-C, HDL-C, VLDL-C, and LDL-C in Type 2 diabetic patients (Kumari, 2010; Nambiar et al., 2010; Ghiridhari et al., 2011). Leaf powder capsules (4 g) significantly increased the secretion of insulin in healthy subjects (Anthanont et al., 2016).

*M. oleifera* seed extract reduced lipid peroxidation and increased antioxidant enzyme activity in streptozotocin-induced mice (Al-Malki and El Rabey, 2015). It also reduced the immunoglobin IgG and IgA activity of the mice which correlated to the reduction of IL-6 that is responsible for glucose homeostasis and pancreatic beta cell activity. The seed extract normalized the activity of both damaged kidneys and pancreases without changing the pathology of the mice. It was reported that the bioactive compounds involved were quercetin (**3**), kaempferol (**8**), glucomoringin (**30**), and chlorogenic acid (**54**) and that they had various biological activities such as their anticancer, antioxidant, hypotensive, anti-inflammatory, hypoglycemic, and antidyslipidemic effects. Other than these compounds, isothiocyanates (**34–37**) had also been reported to show antidiabetic activity (Waterman et al., 2015). A study reported that benzylamine (**108**) isolated from *M. oleifera* reduced the plasma cholesterol, body weight gain, hyperglycemic responses, and fasting blood glucose levels of high fat diet-induced mice (Iffiu-Soltesz et al., 2010).

Barbagallo et al. (2016) reported that *M. oleifera* reduced IL6 expression and had protective effect toward adipocytes by inducing the expression of heme-oxygenase-1. The extract increased IRS-1 gene expression which is responsible for insulin resistance and a shortage of which causes type 2 diabetes. In addition, the extract also induced thermogenesis during the differentiation of adipose tissue by upregulating the activity of mediators of thermogenesis, specifically the uncoupling protein, PPARα, sirtuin 1, and coactivator 1 α. *M. oleifera* extract (100 μg/mL) showed the same behavior as 0.4 μg/mL pravastatin in inhibiting HMG-CoA reductase and decreasing cholesterol biosynthesis (Duangjai et al., 2011). *M. oleifera* also displayed synergistic effects with sitagliptin showing antihyperglycemic activity and delaying lenticular opacity in diabetic rats (Olurishe et al., 2016).

A different reaction of HDL level was observed between normal rabbits and hypercholesterolemic rabbits fed with *M. oleifera*. *M. oleifera* decreased HDL levels in normal rabbits but increased HDL levels in hypercholesterolemic rabbits (Mehta et al., 2003; Nunthanawanich et al., 2016). The lipid profiles of the aorta, heart, and liver were reduced in the hypercholesterolemic rabbits.

### Antifertility

Mekonnen (2002) reported that an ethanolic extract of *M. stenopetala* leaves reduced fertility by 73.3%. The extract exhibited oxytocic activity on guinea pig and mouse uteri. The extract also increased the smooth muscle of the mice uteri that might lead to contraction, thus rejecting implantation. *M. concanensis* stem bark also inhibited implantation by 46% using 400 mg/kg of the extract (Ravichandiran et al., 2007). The reaction was solvent-dependent. The ethyl acetate fraction showed the lowest anti-implantation activity in contrast to chloroform, petroleum ether, and an ethanol extract.

It was reported that *M. oleifera* leaf extract aborted 100% of implantation in seven rats (Sethi et al., 1988). The extract was administered after 5–10 days after mating. The aqueous root extract of the plant did not promote a favorable condition of the uterus for implantation of the fertilized eggs (Prakash et al., 1987). A high dose of the root extract, 600 mg/kg, had anti-progestational activity, preventing the formation of the deciduoma in rats (Shukla et al., 1988). It also reduced the protein concentration for the formation of the uterus (Prakash et al., 1988).

### Anti-inflammation

A study reported that an ethanolic extract of the *M. concanensis* flower and fruit inhibited inflammation by 78.4 and 44.08%, respectively (Rao et al., 2008; Jayabharathi and Chitra, 2011). An extract of the aerial part of *M. peregrina* decreased the effect of peritorial inflammation and reduced the permeability of small blood vessels (Elbatran et al., 2005). Ethanolic and aqueous extracts of *M. peregrina* seeds inhibited fresh egg albumin-induced acute inflammation in rats at doses of 100–300 mg/kg p.o (Koheil et al., 2011).

The major anti-inflammation mechanism reported for *M. oleifera* was the inhibition of the NF-κB pathway. Four fractions of *M. oleifera* leaf (hexane, chloroform, ethyl acetate, and butanol) reduced IL-1β, IL-6, PGE2, TNF-α, and nitric oxide production in LPS macrophages (Arulselvan et al., 2016). Among the fractions, ethyl acetate possessed the strongest inhibition effects and was therefore further analyzed. The extract blocked the nuclear translocation of NF-κB and increased inhibitor κB expression, which was also observed in a fruit extract of *M. oleifera*. Higher concentrations (500 and 1,000 μg/mL), especially from chloroform fraction, were reported to be cytotoxic. Kooltheat et al. (2014) reported that an ethyl acetate extract of *M. oleifera* leaves suppressed the expression of RelA. A hydro ethanolic flower extract also reduced the activity of inflammatory mediators and proinflammatory cytokines such as PGE2, IL-6, IL-1β, TNF-α, NF-κB, iNOS, NO, and COX2 in LPS-induced RAW264.7 macrophages (Tan et al., 2015). In addition, the extract increased the activity of the anti-inflammatory cytokines IL-10 and 1κB-α. Among the different parts of *M. oleifera*, the fruit showed the highest activity in reducing NO release induced by LPS in RAW264.7 cells (Lee et al., 2013).

Sashidara et al. (2009) isolated aurantiamide acetate (**69**) and 1,3-dibenzyl urea (**74**) from *M. oleifera* roots that exhibited inhibition toward IL-2 activity. Aurantiamide acetate (**69**) also inhibited TNF-α activity. 4-[(α-*L*-rhamnosyloxy)benzyl]isothiocyanate (**34**) and 4-[(4′-*O*-acetyl-α-L-rhamnosyloxy)benzyl]isothiocyanate (**37**) from *M. oleifera* leaves displayed anti-inflammatory activity by regulating IL-1β and iNOS expression in addition to reducing the production and expression of inflammatory markers in RAW macrophages (Waterman et al., 2014). 4-[(α-*L*-rhamnosyloxy)benzyl]isothiocyanate (**34**) exhibited anti-inflammatory and antioxidant activity against cerebral tissue damage induced by cerebral ischemia reperfusion in rats (Galuppo et al., 2015b). The compound reduced iNOS, phospho-ERK p42/44, TNF-α, MMP-9, p-selectin, and NFκBp65 nuclear translocation/IκB-alpha cytosolic degradation. A study also noted that 4-[(2′-*O*-acetyl-α-*L*-rhamnosyloxy) benzyl]isothiocyanate (**35**) suppressed COX-2 and iNOS activity in addition to inhibiting the expression of NO in RAW264.7 mouse macrophage cells induced by LPS (Park et al., 2011). The compound inhibited NF-κB activation, the phosphorylation of ERK1/2, IκBα the phosphorylation of IKKα/β, and increase degradation of IκBα. The isothiocyanate showed stronger activity than other isothiocyanates such as benzyl isothiocyanate and sulforaphane. 4-[(α-*L*-rhamnosyloxy)benzyl]isothiocyanate (**34**), 4-[(2′-*O*-acetyl-α*-L-*rhamnosyloxy)benzyl]isothiocyanate (**35**), 4-[(3′-*O*-acetyl-α-*L*-rhamnosyloxy)benzyl] isothiocyanate (**36**), and 4-[(4′-*O*-acetyl-α-*L*-rhamnosyloxy)benzyl] isothiocyanate (**37**) inhibited the production of NO (Cheenpracha et al., 2010).

Galuppo et al. (2014) reported that 4-[(α-*L*-rhamnosyloxy) benzyl] isothiocyanate (**34**) exhibited anti-inflammatory activity toward multiple sclerosis cascades. The compound specifically reduced the imbalance of Bax/BCl-2 and inhibited TNF-α activity on myelin oligodendrocyte glycoprotein 35–55-induced C57B1/G male mice. The compound also inhibited GSK3β levels and normalized the Wnt-β-catenin pathway in experimental autoimmune encephalomyelitis mice (Giacoppo et al., 2016). The compound attenuated apoptosis in addition to suppressing PPARγ activation. 4-[(α-*L*-rhamnosyloxy) benzyl]isothiocyanate (**34**), together with α-cyclodextrin, inhibited the phosphorylation of p38 and Akt in LPS-induced inflammation in RAW 264.7 macrophage cells (Giacoppo et al., 2017). Another study reported that quercetin (**3**) and kaempferol (**8**) inhibited transcription 1(STAT-1) (Tan et al., 2015).

A study reported that a methanol extract of *M. oleifera* leaves reduced the edematogenic effect of carrageenan-induced and histamine-induced paw edema (Adedapo et al., 2015). The extract reduced the number of writhes of mice induced by acetic acid. The analgesic activity of the extract was recorded to be higher than that of the reference drug, indomethacin, at doses of 100 and 200 mg/kg. The inflammatory response after administration of an *M. oleifera* ethanolic leaf extract was observed on atopic dermatitis mice and human keratinocytes (Choi et al., 2016). The extract reduced mannose receptor mRNA, retinoic acid-related orphan receptor γT, and thymic stromal lymphopoietin expression in ear tissue. From an *in vitro* assay, it was observed that the extract reduced mitogen-activated protein kinases, CCL17, IL-6 pro-inflammatory cytokine-related mRNA, TNF-α, and IL-1β expression. *M. oleifera* pod extract also inhibited the elevation of protein levels and mRNA of cyclooxygenease-2, TNF-α, IL-*6*, and iNOS by inhibiting the phosphorylation of mitogen-activated protein kinases and κB proteins (Muangnoi et al., 2012).

*M. oleifera* hydroethanolic and methanolic leaf extracts improved the cellular and humoral immunity of normal and immunosuppressed mice in a dose-dependent manner (Gupta et al., 2010; Nfambi et al., 2015). The extract increased the phagocytic index, weight of the thymus and spleen, antibody titer, and white blood cell and neutrophil concentration. An ethanolic extract of *M. oleifera* seeds inhibited the reaction of delayed-type hypersensitivity by reducing mean foot pad thickness on mice (Mahajan and Mehta, 2010). The immunosuppressive activity of the extract was observed by its ability to down-regulate phagocytosis by macrophages. The ethanolic extract of the seeds also reduced white blood cell, and leukocyte concentration which usually leads to an immunity reaction. It increased paw edema which typically resulted in type IV hypersensitivity occurring. Methanolic extracts of *M. oleifera* leaves exhibited analgesic effects by reducing mechanical allodynia and thermal hyperalgesia in Freund's adjuvant arthritis-induced rats (Manaheji et al., 2011). In contrast, *M. oleifera* methanolic root extracts only reduced thermal hyperalgesia in the rats. The activity of the root and leaf extracts were comparable with indomethacin activity. In addition, a combination of root and leaf extracts showed higher reduction of thermal hyperalgesia in lower doses. An ethanol extract of *M. oleifera* leaves and its major compounds, quercetin-3-*O*-glucoside **(18**), kaempferol-3-*O*-glucoside (**21**), and crypto chlorogenic acid (**55**), showed anti-inflammatory activity, inhibiting the migration and chemotactic oxidation of polymorphonuclear leukocytes (Vongsak et al., 2013).

*Moringa* species also inhibited ulcers induced by non-steroidal anti-inflammatory drugs. *M. oleifera* extract reduced gastric lesions from acetylsalicylic acid, serotonin, and indomethacin (Pal et al., 1995), while an extract of the aerial part of *M. peregrina* reduced gastric lesions from indomethacin (Elbatran et al., 2005). A hydroalcoholic extract of *M. oleifera* seeds reduced the lesion severity and size of acute colitis induced by acetic-acid in rats (Minaiyan et al., 2014). The extract also reduced the activity of TNF-α, IL-4, IL-6, and myeloperoxide which are usually responsible for inflammatory bowel diseases. Debnath and Guha (2007) reported that an *M. oleifera* aqueous leaf extract reduced the mean ulcer index in addition to increasing 5-HT concentration and enterochromaffin cell concentration. The extract protected against ulcer formation in aspirin-induced rats by stimulating enterochromaffin cells in the gastrointestinal tract via 5-HT_3_ receptors (Debnath et al., 2011).

The ethyl acetate fraction of *M. oleifera* leaves obtained from a hydroethanolic extract of the plant increased normal human dermal fibroblast migration and cell proliferation (Gothai et al., 2016). It was observed that a higher dose, more than 125 μg/mL, reduced cell proliferation. High concentrations of phenols in the extract might induced caspase and apoptosis. The migration observed also displayed a 9% better activity than the positive control, allantoin. The leaf ethyl acetate extract contained 4-[(2′-*O*-acetyl-α-*L*-rhamnosyloxy)benzyl]isothiocyanate (**35**) and 4-[(3′-*O*-acetyl-α-*L*-rhamnosyloxy)benzyl]isothiocyanate (**36**), both of which have anti-inflammatory activity. Muhammad et al. (2016) reported that a leaf extract of *M. oleifera* proliferated tissue cells, hence reducing the wound size of diabetic foot ulcers. It downregulated the activity of the inflammatory mediators TNF-α, IL-1β, IL-6, iNOS, and COX-2. The extract also increased angiogenesis activity by reducing the time needed for the wound healing phase, in which vascular endothelial growth factor activity occurs. The study reported that the bioactive compound for this activity was vicenin-2 (**17**). An aqueous fraction of *M. oleifera* leaves reduced scar areas and increased the closure rates of wounds in addition to increasing the granuloma and skin breaking strength, granuloma dry weight, and hydroxyproline content of albino rats (Rathi et al., 2006). *M. oleifera* protease activity from leaf and root aqueous extracts exhibited proteolytic, fibrinolytic, and fibrinogenolytic activity on blood clotting (Satish et al., 2012). The protease showed similar activity to plasmin and thrombin. The protease also attenuated apoptosis in addition to suppressing PPAR gamma activation. Bhatnagar et al. (2013) reported that an *M. oleifera* seed extract, together with *Acacia arabica* biopolymers, had potential as a wound dressing material. In addition to being good antimicrobial substances, the biopolymers were biodegradable and could absorb water from 415 to 935%. They also shortened the time taken to activate partial prothrombin and thromboplastin.

### Antihypertension

Safaeian et al. (2015) showed that an *M. peregrina* leaf extract, administered before and during the administration of dexamethasone to rats, prevented their systolic blood pressure from increased but did not reduce already increased systolic blood pressure. The bioactive dose, 400 mg/kg, showed low but significant antihypertensive activity. The compounds that were reported responsible for this activity were quercetin (**3**), apigenin (**9**), and lupeol (**82**) which had been previously reported to possess antihypertension activity. Safaeian et al. (2015) mentioned that the plants used were obtained during summer and this might affect the concentration of bioactive compounds in the plant. This correlated to a study that observed that the total phenolics in *M. oleifera* harvested in winter was higher than that in plants harvested in summer (Shih et al., 2011).

The vasodilatory activity of an *M. stenopetala* leaf crude extract and fraction were reported by Geleta et al. (2016a). The activity was observed in the thoracic aorta of guinea pigs that had been induced by various vascular contraction agents. Aqueous and 70% ethanol crude extracts of the leaves reduced the vascular contraction induced by potassium chloride, methylene blue, epinephrine, and glibenclamide, in addition to inhibiting the increment of fructose-induced blood pressure in rats in a dose-dependent manner. The extract possessed alkaloids and glycosides that might have cause stronger activity than observed with the fractions. In the study, the extract had the greatest activity toward vascular contraction induced by potassium chloride either when the thoracic aorta was attached to the endothelium, or not. Geleta et al. (2016a) suggested that the extract blocked Ca^2+^ channels during this activity. The endothelium might have stimulated relaxing factors that increased the extract's activity.

A study reported that an ethanol extract of *M. oleifera* leaves reduced pulmonary arterial blood pressure immediately after administration of monocrotaline to rats (Chen et al., 2012). As pulmonary hypertension is usually related to increased reactive oxygen species in the system, the antioxidant activity of the extract that increased SOD activity might be the largest contribution toward its antihypertension activity on pulmonary hypertension. The bioactive compounds reported in this study were niazirin (**97**) and niaziridin (**98**). *M. oleifera* seeds also exhibited cardioprotective activity in spontaneous hypertensive rats (Randriamboavonjy et al., 2016). The extract increased cardiac diastolic function and reduced nocturnal heart rate without modifying the blood pressure of the rats. It also reduced fibrosis and the thickness of the left ventricular relative, interseptal, and anterior walls, in addition to reducing cardiac triglyceride levels. Plasmatic prostacyclin and PPAR-α and γ, activity was also increased by the extract. The study concluded that the extract exhibited antifibrotic and antihypertrophic activity that helped protect cardiac function in hypertension rats.

In addition, it was reported that the antioxidant activity of an *M. oleifera* leaf butanolic extract helped to reduce cardiac necrosis and oxidative stress in isoproterenol-induced rats (Panda, 2015). The extract reduced cardiac lipid peroxidation and increased cardiac antioxidants. The free radicals in the cardiac region were inhibited by the extract with IC_50_-values of 19.92 ± 1.19 μg/mL which was comparable with quercetin (**3**) activity (IC_50_-value of 19.95 ± 1.17 μg/mL). The extract also reduced inflammation and necrosis to almost normal myofibrillar structure. *N*,α-*L*-rhamnopyranosyl vincosamide (**67**) exhibited cardioprotective activity by reducing the level of serum cardiac markers and myocardial necrosis in addition to normalizing the cardiac histology of isoproterenol-induced rats (Panda et al., 2013).

### Antispasmodic

Hydro alcoholic extracts of *M. peregrina* leaves and seeds were investigated for their antispasmodic activity on ileum spasms (Sadraei et al., 2015). The leaves inhibited ileum spasms induced by potassium chlorine (IC_50_: 439 ± 108 μg/mL), electrical field stimulation (IC_50_: 314 ± 92 μg/mL), and acetylcholine (IC_50_: 365 ± 61 μg/mL). It was observed that the seed extract displayed better inhibition than the leaves against ileum spasms induced by potassium chloride (IC_50_: 87 ± 18 μg/mL), electrical field stimulation (IC_50_: 230 ± 51 μg/mL), and acetylcholine (IC_50_: 118 ± 18 μg/mL). The aqueous extract of *M. oleifera* seeds exhibited higher inhibition activity against acetylcholine-induced contractions (ED_50_ of 65.6 mg/mL) (Caceres et al., 1992).

### Others

A study reported that *M. oleifera* exhibited antidepressant activity (Kaur et al., 2015). The crude extract showed positive results in the forced swim test, tail suspension test, and locomotor activity test. The activity was enhanced with co-administration of the SSRI depression drug, fluoxetine. In addition, high doses of the extract, particularly 2,000 mg/kg, did not exert toxicity on the tested mice. Akanni et al. (2014) reported that an ethanol extract of *M. oleifera* leaves exhibited antileukemic activity against benzene-induced leukemic Wistar rats. The extract normalized the leukemic condition, increased GSH, and reduced MDA levels in the rats. Galuppo et al. (2015a) reported that isothiocyanate, specifically 4-[(α-*L*-rhamnosyloxy)-benzyl] isothiocyanate (**34**), isolated from *M. oleifera* delayed ALS development. The compound reduced PARP-1 activity in addition to promoting Nrf-2 activity. The study suggested that the isothiocyanate interfered with motor neuron degeneration and ALS development in the SOD1 rats. Upon administration of 125 mg/kg *M. oleifera* leaf extract, rats' antibody against the Salmonella typhimurium “O” antigen increased to 50%. The extract also increased the concentration of serum immunoglobulins, white blood cells, and neutrophils, hence increasing the humoral immune response of the rats (Jayanthi et al., 2015).

## Toxicity

Awodele et al. (2012) reported that an aqueous extract of *M. oleifera* leaves did not produce any mortality in Wistar albino mice at orally administered doses of up to 6,400 mg/kg. Higher doses (3,200 and 6,400 mg/kg) did triggered dullness and reduced locomotion in the rats. There was no significant difference observed on the rats' sperm quality, or on their hematological, histological, and biochemical parameters. The LD_50_-value was determined to be 1,585 mg/kg. In another study, it was reported that high doses of *M. oleifera* leaves (3,000 mg/kg) caused the presence of micro nucleated polychromatic erythrocytes in the femur bone marrow of Sprague-Dawley rats (Asare et al., 2012). The study reported that doses of more than 3,000 mg/kg caused acute toxicity and increased the urea levels of the rats. The study reported that this was caused by high concentrations of nitrogenous compounds in the *M. oleifera*, potentially from proteins.

## Conclusion

Various research has been conducted to evaluate the traditional uses of *Moringa* species and all of the research supported the traditional claims. However, there are still an abundance of traditional uses that have not been evaluated, especially in species other than *M. oleifera* and *M. stenopetala*. Hence, further research is needed to exploit the many uses of *Moringa* species.

## Author contributions

NA obtained the literatures and wrote the manuscript while KH and EK gave ideas and edited the manuscript.

### Conflict of interest statement

The authors declare that the research was conducted in the absence of any commercial or financial relationships that could be construed as a potential conflict of interest.
